# Bright luminescence from pure DNA-curcumin–based phosphors for bio hybrid light-emitting diodes

**DOI:** 10.1038/srep32306

**Published:** 2016-08-30

**Authors:** M. Siva Pratap Reddy, Chinho Park

**Affiliations:** 1 LED-IT Fusion Technology Research Center, Yeungnam University, Gyeongsan 38541, Republic of Korea; 2 School of Chemical Engineering, Yeungnam University, Gyeongsan 38541, Republic of Korea

## Abstract

Recently, significant advances have occurred in the development of phosphors for bio hybrid light-emitting diodes (Bio-HLEDs), which have created brighter, metal-free, rare-earth phosphor-free, eco-friendly, and cost-competitive features for visible light emission. Here, we demonstrate an original approach using bioinspired phosphors in Bio-HLEDs based on natural deoxyribonucleic acid (DNA)-curcumin complexes with cetyltrimethylammonium (CTMA) in bio-crystalline form. The curcumin chromophore was bound to the DNA double helix structure as observed using field emission tunnelling electron microscopy (FE-TEM). Efficient luminescence occurred due to tightly bound curcumin chromophore to DNA duplex. Bio-HLED shows low luminous drop rate of 0.0551 s^−1^. Moreover, the solid bio-crystals confined the activating bright luminescence with a quantum yield of 62%, thereby overcoming aggregation-induced quenching effect. The results of this study herald the development of commercially viable large-scale hybrid light applications that are environmentally benign.

The use of rare-earth phosphors in blue light-emitting diode (BLED) technology has several disadvantages^1^,^2^, such as the (i) metallic or inorganic (ii) cost-intensive and (iii) toxic nature of these compounds. These limitations should be overcome to produce BLED-based rare-earth phosphor free LEDs (RPF-LEDs). Numerous quantum dot-based technical methodologies for developing RPF-LEDs have been successfully demonstrated^3^,^4^,^5^. However, most of them involve complicated multi-step procedures, which could lead to secondary issues such as increased manufacturing cost, reduced target yield, and environmental problems. Therefore, handling the relationship between environmental factors associated with new methodologies and possible cost escalation is an on-going challenge.

Recently, biomaterials including DNA, fluorescent proteins, carbon dots, and vegetables have attracted enormous interest because of their interesting luminescence characteristics^6^,^7^,^8^,^9^. Even more interesting, fluorescent proteins have been applied to develop a new device concept called bio hybrid light-emitting diode (Bio-HLED)8. Another alternative in this emerging field is the bioinspired phosphors achieved by the use of DNA as bio-scaffold. The study of bioinspired phosphors achieved by the use of DNA as an optical material was a great success^10^. Some researchers have explored various chromophores such as coumarin 102, 4-[4-(dimethylamino) styryl]-1-docosylpyridinium bromide, sulforhodamine, and pyrromethene 567 to DNA for the fabrication of white LEDs (WLEDs) using a fluorescence resonance energy transfer (FRET) technique^11^,^12^. Furthermore, DNA is among the most abundant materials on earth; however, these chromophores are very expensive.

In this study, we propose curcumin as a chromophore for DNA for Bio-HLED fabrication as a very productive and cost-innovative methodology, and demonstrate its bright, stable luminescence. We selected curcumin extract from turmeric as a chromophore, which generates efficient luminescence. Furthermore, curcumin absorbs in the UV to the visible region of the spectrum, producing fluorescence^13^. Curcumin is widely available in nature, obtained via solvent extraction of turmeric (*Curcuma longa* Linn. rhizomes), and a common ingredient in spices, cosmetics, and traditional medicines in Asian countries^14^. To our knowledge, curcumin is the most inexpensive chromophore worldwide. Because of these numerous advantages, we propose a new approach to obtaining brighter and cost-competitive visible light emitters using curcumin as a DNA chromophore.

## Results and Discussion

### Curcumin chromophore binding to DNA duplex

**Figure 1:**
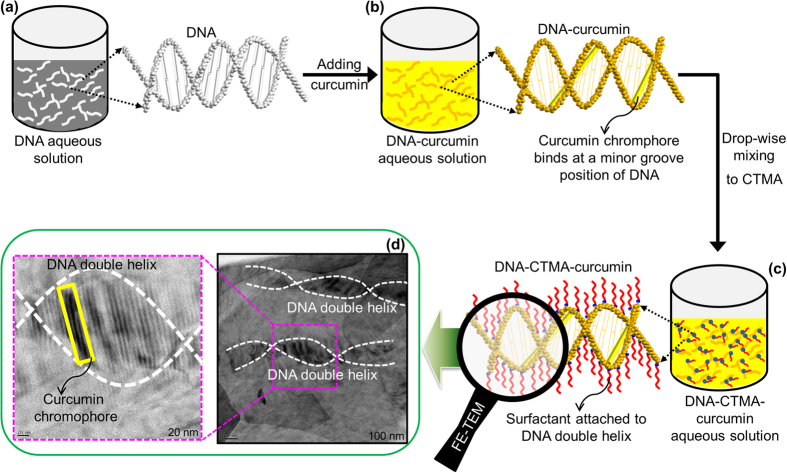
Processing steps or preparation of DNA-CTMA-curcumin aqueous solution. (**a**) DNA (**b**) DNA-curcumin (**c**) DNA-CTMA-curcumin aqueous solutions, and (**d**) FE-TEM images of curcumin chromophore bounds to minor groove position of DNA double helical structure under different magnifications (scale bar, 20 and 100 nm).

The schematic portrayal of the procedure used for the preparation of the DNA-curcumin aqueous solution is illustrated in Fig. 1. In the first step, the DNA powder is dissolved in water (Fig. 1a), followed by the second step, which involves adding curcumin to the aqueous DNA solution, where its nanomolecules specifically bind at minor groove positions of DNA, as clearly shown in Fig. 1b. The chemical structure and minor and major groove positions of DNA with base pairs (adenine, A, thymine, T, cytosine, C, and guanine, G) is perceivably shown in Supplementary Fig. S1. The aqueous DNA-curcumin solution is not suitable for fabricating the device and, therefore, cetyltrimethylammonium (CTMA) was introduced as a surfactant to precipitate the solution into the lipid form as shown in Supplementary Fig. S2. The phosphate-based double helical structure of DNA is negatively charged and is therefore attracted by the positively charged CTMA surfactant, as shown in Fig. 1c. Hence, this is the key process for producing the DNA-curcumin precipitate with CTMA. Verifying the binding of curcumin chromophore to the DNA structure was extremely critical to confirming our data. To achieve this, we compared the representative 3-dimensional binding structures (Fig. 1c) and the identified real images using field emission tunnelling electron microscopy (FE-TEM) images of curcumin chromophore bound to the minor groove position of the DNA duplex (Fig. 1d). Until now, studies have only been able to infer this type of binding information based on spectroscopic analysis^15^,^16^. In fact, curcumin binds to DNA by hydrogen bonding interactions with the minor groove in adenine-thymine (AT) base pairs^16^,^17^. Figure 1d shows the FE-TEM images for the curcumin chromophore binds to DNA duplex with different image resolutions. DNA that bind curcumin chromophore generally exhibit marked changes in absorbance and fluorescence properties compared to their spectral characteristics when free in solution^16^.

### Structural properties

**Figure 2:**
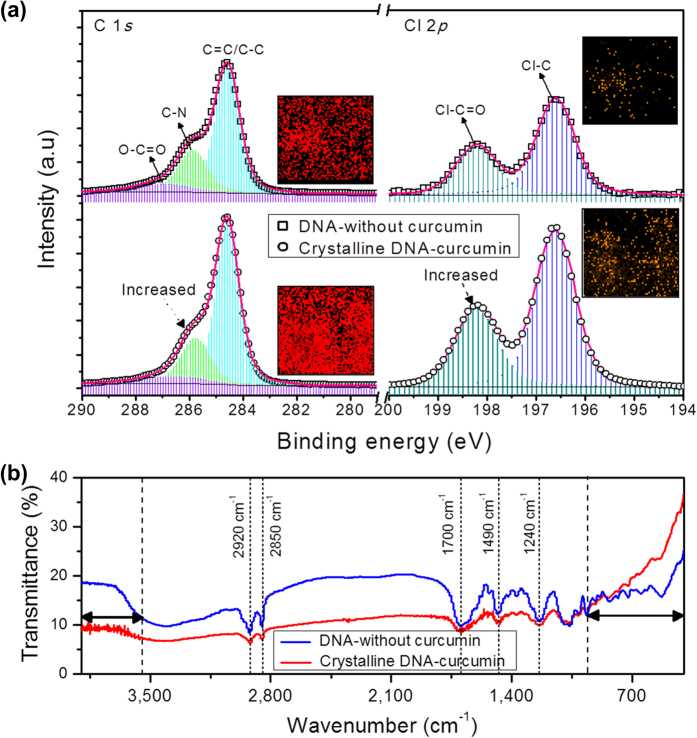
Surface states and stretching of DNA-without curcumin and crystalline DNA-curcumin samples. (**a**) XPS spectra of C 1*s* and Cl 2*p* core level for DNA-without curcumin and crystalline DNA-curcumin. For C 1*s*, cyan lines are C=C/C−C bonding, green lines are C−N bonding, and purple lines are O−C=O bonding, and for Cl 2*p*, blue lines are Cl−C bonding and bright green lines are Cl−C=O bonding. Inset figures show elemental mapping of C (red colour) and Cl (orange colour) in DNA-without curcumin and crystalline DNA-curcumin analysed using the STEM technique. (**b**) FTIR spectra of DNA-without curcumin and crystalline DNA-curcumin samples.

Bright luminescence from unprocessed DNA-curcumin with CTMA solution or the lipid form is difficult to handle because of aggregation induced quenching effect and, therefore, unpractical for use. To reduce quenching effect requires a crystalline form, which we obtained crystalline DNA-CTMA-curcumin complex (‘crystalline DNA-curcumin’ hereafter) using an oven heating process. Then, confirming the crystalline state of crystalline DNA-curcumin was one of the key objectives of this investigation. To achieve this, we performed X-ray photoelectron spectroscopy (XPS) measurement to determine surface states in the crystalline DNA-curcumin, and compared the results to those obtained without curcumin in the DNA-CTMA (‘DNA-without curcumin’ hereafter). Based on the XPS results shown in Fig. 2a, increased peak intensities of the C 1*s* and Cl 2*p* (such as Cl−C=O and Cl−C bond peaks) in the crystalline DNA-curcumin were observed, indicating that the carbonyl structures and halogen content was more in the presence of curcumin than in its absence. The inset of Fig. 2a shows the elemental-mapped images of C and Cl in the crystalline DNA-curcumin and DNA-without curcumin, as analysed by scanning transmission electron microscopy (STEM). The images clearly show that the atomic compositions of C and Cl in the crystalline DNA-curcumin sample were high compared to those for the sample DNA-without curcumin. Further, the elemental spectra of C and Cl in crystalline DNA-curcumin and DNA-without curcumin samples were measured using scanning electron microscopy/energy dispersive X-ray diffraction (SEM/EDX) to confirm the XPS results, as shown in Supplementary Fig. S3. Furthermore, to confirm the carbonyl structures and bond stretches of crystalline DNA-curcumin and DNA-without curcumin samples, we performed Fourier transform infrared spectroscopy (FTIR) measurement as shown in Fig. 2b. Absorption bands at 2,920 cm^−1^ and 2,850 cm^−1^ are asymmetric and symmeric strechings of CH_2_, respecively. In addition, absorption bands around 1,700 cm^−1^, 1,490 cm^−1^, and 1,240 cm^−1^ are streching vibrations of C=O, aromatic ring streching and in-plane bending, and aromatic CCH in-plane bending, respectively. In the absence of curcumin, there was no change in the spectrum compared to that with curcumin, but the wave number region of 3,600 cm^−1^ (free O−H stretching) above and the wave number region of below 950 cm^−1^ (C−O stretching vibrations) observed that the relative intensity changes. These changes indicated that, in the presence of curcumin, the sample transformed into the crystalline state and as well as it contains carbonyl structures. Similar characteristic strechings are identified in pure crystalline curcumin^18^.

**Figure 3:**
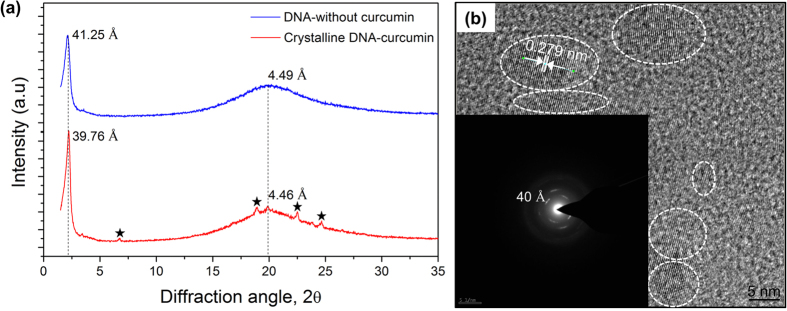
Structural properties of the crystalline DNA-curcumin. (**a**) WAXD spectra of DNA-without curcumin and crystalline DNA-curcumin samples, sharp and broad circular reflection peaks are found at 41.25 Å and 39.76 Å, and 4.49 Å and 4.46 Å which were related to diameter of DNA-without curcumin and crystalline DNA-curcumin complexes and distance between DNA strands, and small sharp peaks (*) are related to crystalline curcumin structure. (**b**) FE-TEM image of crystalline DNA-curcumin sample exhibiting lattice spacing of 0.279 nm (scale bar, 5 nm). Inset figure shows SAED pattern of crystalline DNA-curcumin sample and it revealing that the first-order diffraction spacing of 40 Å.

In order to confirm the double helix structure and crystalline nature of DNA-curcumin sample, wide-angle X-ray diffraction (WAXD) analysis was made. From Fig. 3a, it revealed that the structural anisotropy between the side edge and the crystalline face. A sharp circular reflection peaks found at 39.76 Å and 41.25 Å, which were attributed to the diameter of the crystalline DNA-curcumin and DNA-without curcumin samples. In addition, circular reflection peaks clearly observed from the side edge at about 4.46 Å and 4.49 Å were attributed to the distance between adjacent DNA strands for crystalline DNA-curcumin and DNA-without curcumin. Ghirlando *et al*.^19^ found that the X-ray circular reflection peak at 44 Å for DNA-CTAB complex. As a result, the value distance between DNA strands is 39.76 Å in crystalline DNA-curcumin, a value smaller than DNA-without curcumin (41.25 Å) and previously reported^19^, which implies a more close arrangement of crystalline DNA-curcumin complex. The crystalline nature was confirmed using X-ray diffraction, found a sharp diffraction peaks (2θ = 6.7°, 18.94°, 22.5 °and 24.66°), which indiate that crystalline behaviour. Becauese of these sharp peaks does not found for absence of curcumin sample. Similar behaviour like without sharp peaks were observed in DNA-CTAB complex^19^. Moreover, similar characteristic peaks are identified in crystalline curcumin^20^,^21^, meaning that these sharp peaks occur due to curcumin prescence in DNA-CTMA complex. Based on the FE-TEM results shown in Fig. 3b, to confirm X-ray diffraction results such as structure and crystallnity of DNA-curcumin sample. The FE-TEM reveals that, the DNA-curcumin complex shows crystallinity and the lattice fringes have lattice spacing of 0.279 nm. On the basis of WAXD, and FE-TEM analyses, it can be concluded that, the crystalline DNA-curcumin shows excellent crystalline behaviour. Inset of Fig. 3b shows selected area electron diffraction (SAED) of crystalline DNA-cucrumin sample. SAED reveals that the first-order diffraction spacing of 40 Å. Similar results of SAED pettern was found (41 Å and 40 Å) for DNA-CTAB complex by Yang *et al*.^22^ and Ghirlando *et al*.^19^ The observed result (40 Å) form SAED pattern was well matched with the WAXD measurements (39.76 Å) and previous reports^19^,^22^.

### Optical properties

**Figure 4:**
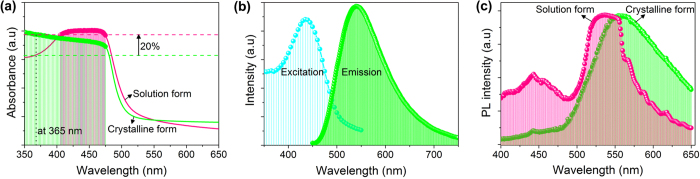
Optical properties of the DNA-curcumin in solution and crystalline form. (**a**) Absorption spectra of DNA-curcumin in solution (pink lines) and crystalline (green lines) forms. (**b**) Fluorescence excitation (cyan lines) and emission (green lines) spectra of DNA-curcumin in solution. (**c**) Photoluminescence spectra of DNA-curcumin in solution (pink lines) and crystalline (green lines) forms.

The optical characteristics including the steady-state absorption spectra of the solution and crystalline forms of the DNA-curcumin are shown in Fig. 4a. The absorption spectral analyses shows that the DNA-curcumin solution and crystalline form had peak absorption wavelength ranges of 405–475 and 350–475 nm, respectively, and the range for the crystalline form was broad. This result indicates that while the DNA-curcumin solution absorbed only in the blue region of the spectrum, the crystalline form also absorbed in the UV region. The detailed description of the advantage of the extended absorption by the crystalline DNA-curcumin form will be discussed later in this paper. To determine the excitation and emission wavelengths of the DNA-curcumin solution, we performed fluorescence measurements (Fig. 4b), and the analysis revealed peak excitation and emission wavelengths of 450 and ~550 nm, respectively. These results clearly suggest that the DNA-curcumin was excited at the blue region and showed a greenish emission in the visible region. The excitation of the DNA-curcumin solution is suitable for commercial purposes due to its excitability in the blue region, because blue-LEDs cost less compared to UV-LEDs. However, despite the low cost of the blue-LEDs, the DNA-curcumin solution used would easily exhibit the activated quenching phenomenon^23^. Therefore, the DNA-curcumin solution may not be suitable for the commercial production of LEDs. Considering the limitations associated with the DNA-curcumin solution, we modified it to its crystalline form using different protocols. Then, to determine the optical emission of the crystalline DNA-curcumin compared with solution form, we performed photoluminescence (PL) measurements, and the results (Fig. 4c) revealed an emission wavelength of ~500–650 nm in the visible region. Further, the crystalline DNA-curcumin showed bright greenish emission, and this behaviour (emission from 500–650 nm) indicated that this form could be tuned to produce yellow emission. In addition, as shown in Supplementary Fig. S4, the excited state lifetime and photoluminescence quantum yield values of curcumin solution and DNA-curcumin solution were 1.65 and 1.81 ns, and 5% and 9%, respectively. The lifetime of the curcumin based solutions were short due to aggregation-induced quenching between biomolecules. Indeed, the vibration of biomolecules is less because of the curcumin chromophore is tightly binding to DNA duplex (Fig. 1b) to emit the luminescence. Even though, this luminescence is not appropriate for generating optical light, and the best strategy for solving this problem is to convert the solution to the crystalline form. The intra bio-molecular motions of the crystalline form are restricted by the crystal design and, compared with the solution, produce more efficient luminescence. The lifetime values of crystalline curcumin, crystalline DNA-curcumin and thick crystalline DNA-curcumin film (in vacuum) were 5.49, 6.01, and 6.79 ns, respectively (Supplementary Fig. S4). The photoluminescence quantum yields were 41% for crystalline curcumin, 62% for crystalline DNA-curcumin, and 67% for thick crystalline DNA-curcumin film (in vacuum).

### Luminous characteristics

**Figure 5:**
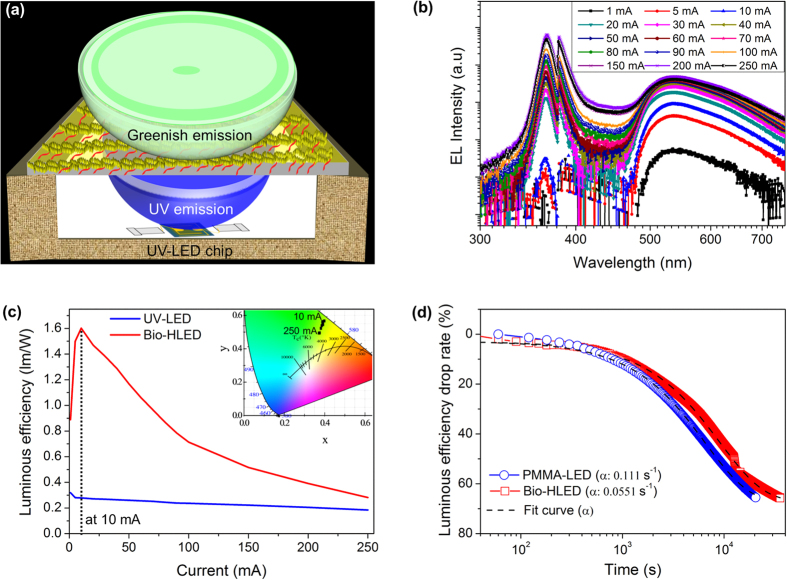
Schematic configuration, optical image, and luminous characteristics of Bio-HLED. (**a**) Configuration of DNA-curcumin–based Bio-HLED. Luminous characteristics of Bio-HLED as a function of injected current from 1–250 mA: (**b**) EL spectra. (**c**) Luminous efficiency including UV-LED comparison, which revealed that Bio-HLED showed bright luminescence at 10 mA compared to normal UV-LED, and inset shows CIE colour co-ordinates of Bio-HLED as a function of injection current from 10–250 mA. (**d**) Time-dependent luminous efficiency drops of PMMA-LED and Bio-HLEDs. Fitting curves are theoretical results calculated from equation (1).

Figure 5 shows the luminous characteristics of the DNA-curcumin–based Bio-HLED. Figure 5a illustrates the schematic diagram of the Bio-HLED and clearly shows the thick crystalline DNA-curcumin film attached to the normal UV-LED. The schematic description of the Bio-HLED device, which consists of a lens coated with crystalline DNA-curcumin and its thick film form are shown in Supplementary Fig. S5. It showed a bright greenish emission when the UV-LED was switched on (Supplementary Fig. S5a,b). The electroluminescence (EL) spectra, Comission Internationale de l′Eclairage (CIE) colour co-ordinates and luminous efficiency of different thickness of crystalline DNA-curcumin thick films were shown in Supplementary Fig. S6 and Supplementary Table 1. It is found that the thickness of crystalline DNA-curcumin thick film was 100 μm shows better than other thickness, due to aforementioned reason further experiment pick out such thickness (100 μm). In addition, the optimum amount of mixing of curcumin (1 g) to DNA at a constant film thickness (100 μm) was confirmed from Supplementary Fig. S7 and Supplementary Table 2. Figure 5b shows the EL characteristics of the Bio-HLED device. In addition, Fig. 5b reveals that the peak of the EL spectrum increased when the current was increased from 1 to 250 mA. The luminous efficiency characteristics as a function of the injected current (from 1 to 250 mA) are shown in Fig. 5c. To investigate the effect of the thick crystalline DNA-curcumin film on the Bio-HLED, we compared it with the results of the normal UV-LED. Moreover, compared the luminous efficiency of same amount (1 g of curcumin to DNA and PMMA, and 100 μm thick film) of the Bio-HLED and PMMA-curcumin (PMMA-LED) thick films were shown in Supplementary Fig. 8. From those results, we conformed that the Bio-HLED shows better luminous efficiency than PMMA-based LED meaning that the using DNA-based approach is beneficial than PMMA approach. The inset of Fig. 5c, the CIE colour co-ordinates of Bio-HLED was measured following injection of currents of 10–250 mA. It is evident, as seen in inset of Fig. 5c, that with increasing current, the colour coordinates transform from greenish (0.39, 0.56) to near yellow emission (0.37, 0.49). Based on these observed characteristics, it would also be possible to develop white light emission using remote luminescence techniques (not shown here). Further, the spatial distribution of the Bio-HLED using angular photon-emission distribution characteristics is shown in Supplementary Fig. 9. Furthermore, the temperature dependent quantum yields of crystalline DNA-curcumin from 27 to 100 °C as shown in Supplementary Fig. 10. It is observed that the quantum yields decreases with increasing temperature. This may be due to the loss of binding interaction between the DNA and curcumin. Also, decrease in quantum yield was not entire depend on the degradation of crystalline DNA-curcumin, it may be depends on the temperature degradation of the UV-LED source. In particular, to evaluate the strength of the binding interactions between DNA and curcumin was confirmed with the help of thermogravimetric analayis (TGA) (Supplementary Fig. 11) in the presence of air. The initial weight loss observed between at 27 °C and 83 °C corresponds well with the loss of water which accounts for 5% of initial weight of crystalline DNA-curcumin. The weight loss observed in the temperature range of 84–300 °C can be attributed to the decomposition of the DNA material suggesting that crystalline DNA-curcumin have higher thermal stability. The high thermal stability and makes crystalline DNA-curcumin suitable for applications in Bio-HLED technology. To investigate the reliability of the luminous characteristics of the Bio-HLEDs, we verified the luminous efficiency drops using an injection current of 10 mA at 27 °C and compared those results to PMMA-LED. Figure 5d shows the plots of the drop rate (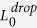) of the luminous efficiency (*L*_0_) for the PMMA-LED and Bio-HLED as a function of the time. The 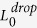 can be expressed as follows:


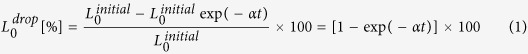


where 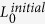 is the initial luminous efficiency measured at the current injection time of 0.1 s, α is the drop rate, and t is the measured time. The α of the Bio-HLED is around 2 times lower than that of the PMMA-LED, indicating that it has a lower drop rate, in this first report of such a pure DNA-curcumin**–**based phosphor material compared with other DNA-based materials^11^,^12^. Further, the degradation of the EL spectra of Bio-HLED over a time is shown in Supplementary Fig. 12. Therefore, the Bio-HLED exhibits a long lifetime with stable characteristics at high injection currents, due to the bright luminescence in the crystalline structure without any aggregation-induced effects. Based on these bright luminescence characteristics, we proposed that crystalline DNA-curcumin is an extremely promising phosphor that can provide a significant source of phosphors to meet future requirements for developing solid-state LEDs. Furthermore, its advantageous properties include absence of metal and rare-earth phosphors, as well as its eco-friendliness, long-life, and cost-competitiveness.

## Conclusions

In conclusion, we used the natural-based material, curcumin as a chromophore material for DNA for producing bright luminescence in a novel and hybrid innovation. The proposed application of curcumin provides a low-cost and convenient approach to prepare phosphors. We use state-of-the-art FE-TEM to observe the images of the curcumin chromophore binding to the minor groove position of the DNA duplex. Moreover, using the curcumin-based phosphors in the Bio-HLED device produced a superior performance with a quantum yield of 62%. Therefore, crystalline DNA-curcumin is an extremely promising phosphor material as confirmed by our study, and we further highlighted numerous advantages in our theoretical explanations. These advantages include its high device performance and excellent reliability, which demonstrate the revolutionary nature of the bio-related optoelectronics, bio-imaging, bio-sensing and data security employing the biomaterial curcumin as a tool for future generations.

## Methods

### Experimental description

For this study, frozen DNA (extracted from salmon fish sperm) powder and the cationic surfactant CTMA were purchased from Sigma-Aldrich. To obtain the DNA-curcumin–based phosphors, we used the following experimental protocol. The DNA powder (5 g) was mixed with 500 ml of de-ionized water in a glass container and stirred for 4 h. Then, 1 g of curcumin (purchased from an Indian supermarket, extracted from turmeric) was mixed with 1 ml of dimethyl sulfoxide (DMSO), the mixture was added to the aqueous solution of DNA, and this mixture was stirred using a magnetic stirrer for 4 h. Then, the yellow coloured aqueous solution of DNA-curcumin obtained was treated with CTMA using an ion-exchange reaction. This reaction induced the substitution of Na ions in the CTMA associated with the DNA base pairs, leading to the precipitation of CTMA and DNA-curcumin at the bottom of the glass container in the resulting aqueous NaCl solution. The DNA-CTMA-curcumin precipitate was separated and oven-dried for 2 h at 60 °C. The DNA-CTMA-curcumin precipitate was separated and oven-dried for 2 h at 60 °C in presence of air. During oven-dried process, liquid content in the precipitate was slowly evaporated. Then, the DNA-CTMA-curcumin precipitate was converted to the bio-crystalline form, which was scraped and powdered, and the practical schematic illustration of the experimental protocol is shown in Supplementary Fig. 2. First method of device technique was crystalline DNA-curcumin solution was coated on lens. Second technique was DNA-CTMA-curcumin convert into thick film form by doctor blade method using glass slides and then kept in oven, after oven-dried, thick film pilled-off from the glass slide.

### Characterization techniques

The optical absorption and fluorescence spectra were recorded using a Cary5000 spectrometer and a fluorescence spectrophotometer (Hitachi F7000), and excited state lifetimes were verified with FL920 (Edinburgh Instruments). The DNA structure was examined using a high resolution FE-TEM (Tecnai G^2^ F20 S-TWIN) and the elemental mapping images were analysed using STEM. Furthermore, XPS (Thermo-Fisher Scientific, K-Alpha) using Al Kα radiation (1,486.6 eV) was used to examine the surface characteristics. The elemental spectrum was found using SEM/EDX (SEM Hitachi S-4200) microscope. The structural properties were characterized by WAXD (Rigaku, D/Max-2500) using Cu Kα radiation (λ = 0.154 nm). FTIR spectra were recorded with a spectral range of 3,900–500 cm^−1^ using Thermo Scientific, Nicolet 6700 spectrometer. The photoluminescence characteristics were investigated using a He-Cd laser (power, 20 mW) at a wavelength of 325 nm with a SPEX 750, HORIBA with temperature adjustable controller. TGA analysis was carried out up to 600 °C at a heating rate of 10 °C/min under air on a SDT Q600. For Bio-HLED measurements used normal UV-LED was manufactured from Nichia (Model: NCSU033B) with an emission wavelength of 365 nm and power, 450 mW. The luminous characteristics including the electroluminescence, luminous efficiency, quantum yield, and CIE colour coordinates were examined using an integrated sphere LED measurement system (PIMACS, NeoLight IS500) consisting of a semiconductor parameter analyser, an optical power sensor, and a temperature controller. The angular spatial photon-distribution characteristics were measured by a goniometer on a wafer level LED measurement system (OPI-160).

## Additional Information

**How to cite this article**: Reddy, M. S. P. and Park, C. Bright luminescence from pure DNA-curcumin-based phosphors for bio hybrid light-emitting diodes. *Sci. Rep.* **6**, 32306; doi: 10.1038/srep32306 (2016).

## Supplementary Material

Supplementary Information
