# Supplementary material for: Bright luminescence from pure DNA-curcumin–based phosphors for bio hybrid light-emitting diodes
Source: Sci Rep. 2016 Aug 30;6:32306. doi: 10.1038/srep32306 (PMC5004171; doi:10.1038/srep32306)
Supplement: Supplementary Information [file srep32306-s1.pdf]

SUPPLEMTARY INFORMATION

Bright luminescence from pure DNA-curcumin–based phosphors for bio hybrid light-emitting diodes

M. Siva Pratap Reddy[1] and Chinho Park[1,2]*

[1]*LED-IT Fusion Technology Research Center, Yeungnam University, Gyeongsan 38541, Republic of Korea*
[2]*School of Chemical Engineering, Yeungnam University, Gyeongsan 38541, Republic of Korea*

*Email: chpark@ynu.ac.kr (C. Park)
TEL : +82-53-810-2522
FAX : +82-53-810-4613

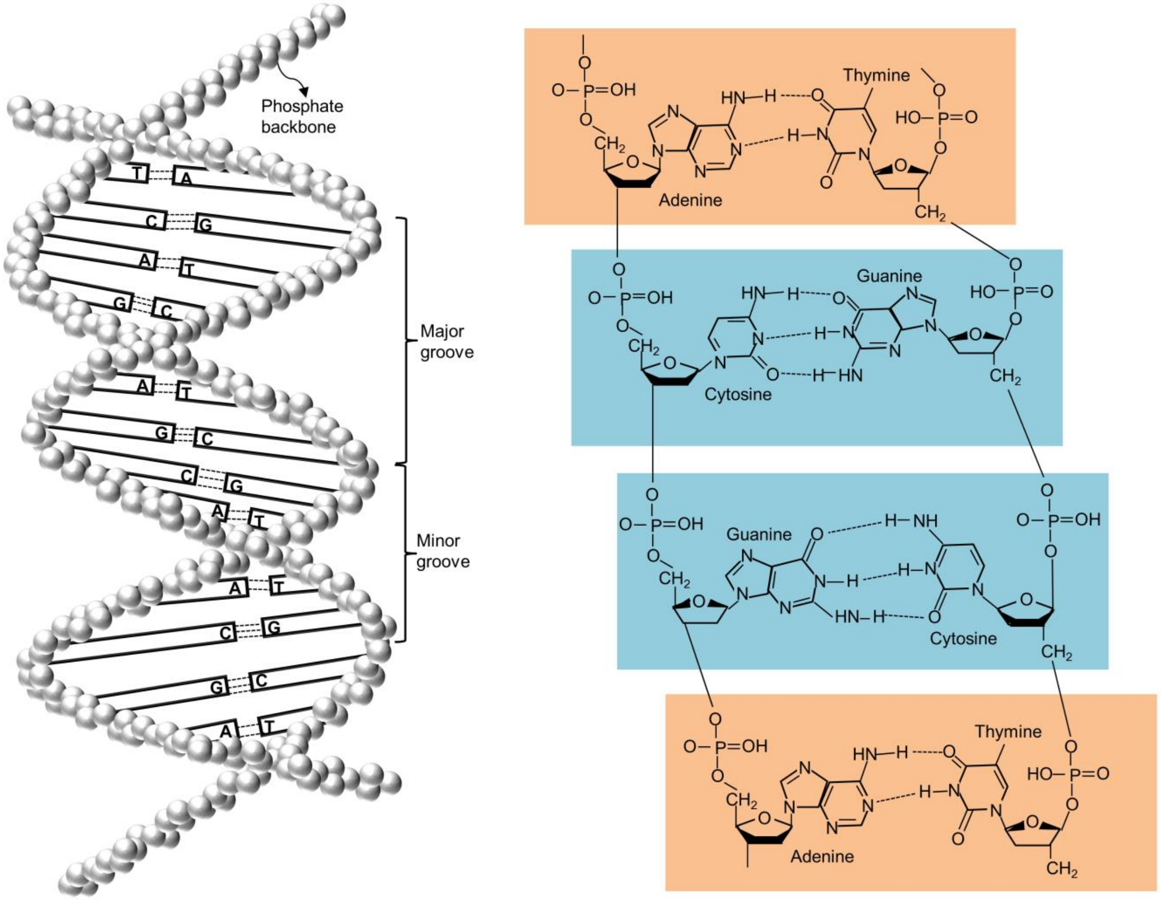

Supplementary Figure S1. Schematic chemical structure and minor and major groove positions of DNA (contains sugar phosphate backbone) with base pairs of adenine, A, thymine, T, cytosine, C, and guanine, G.

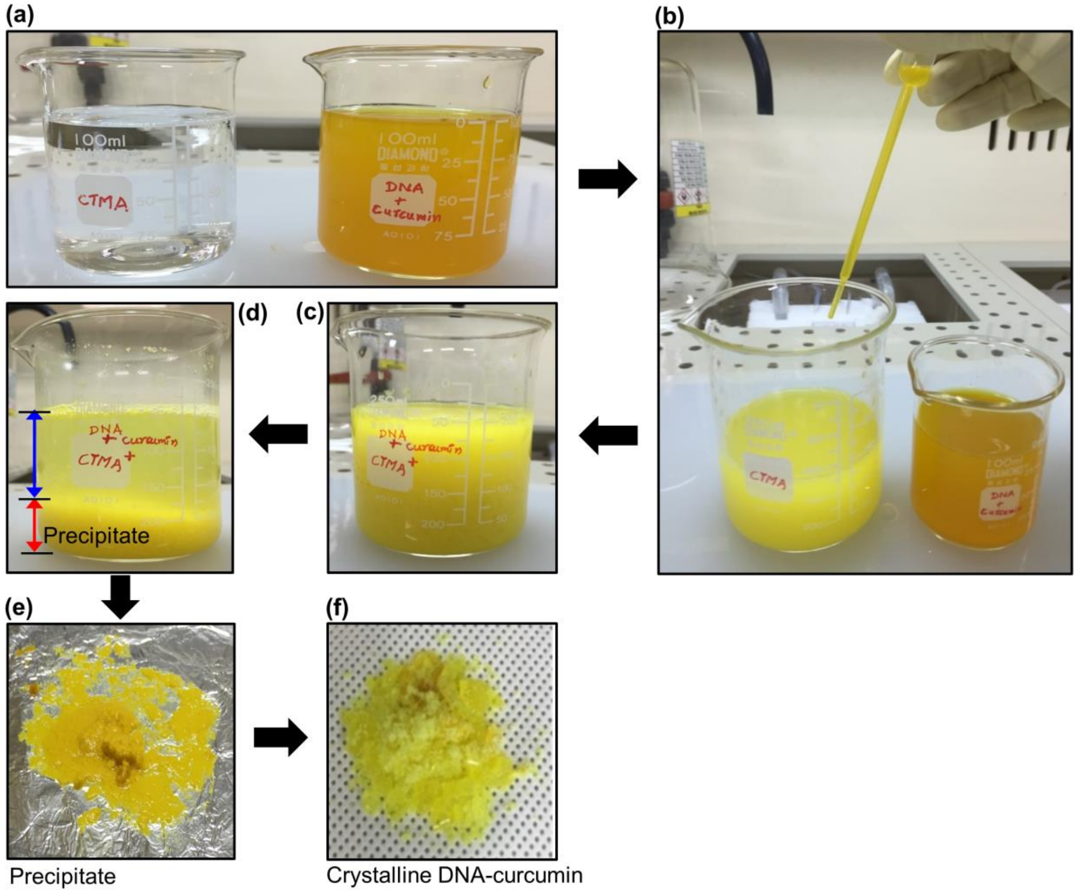

Supplementary Figure 2. Pictorial representation of experiment steps for DNA-CTMA-curcumin crystalline powder preparation protocols (a) Combination of CTMA and aqueous solution of DNA-curcumin. (b) Mixing of aqueous DNA-curcumin solution in CTMA. (c) Mixed aqueous DNA-curcumin-CTMA solution. (d) DNA-curcumin-CTMA precipitate out from the solution. (e) Separation of precipitate from solution. (f) Finally, Crystalline DNA-CTMA-curcumin is obtained by oven-drying for 2 h at 60 ℃.

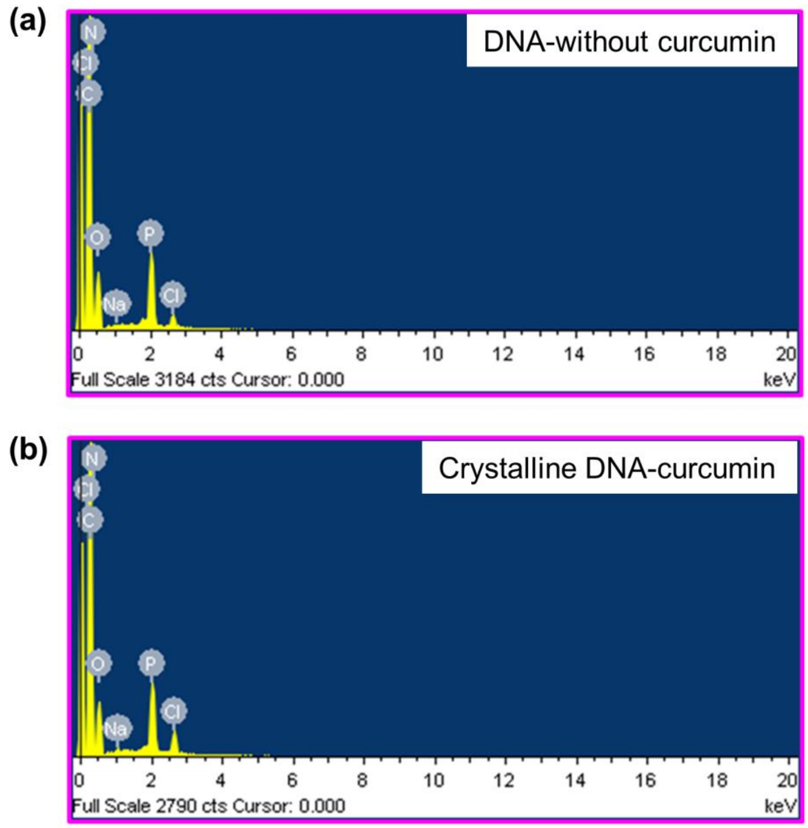

Supplementary Figure S3. Elemental spectra of (a) DNA-without curcumin and (b) crystalline DNA-curcumin obtained using energy dispersive X-ray spectroscopy (EDX).

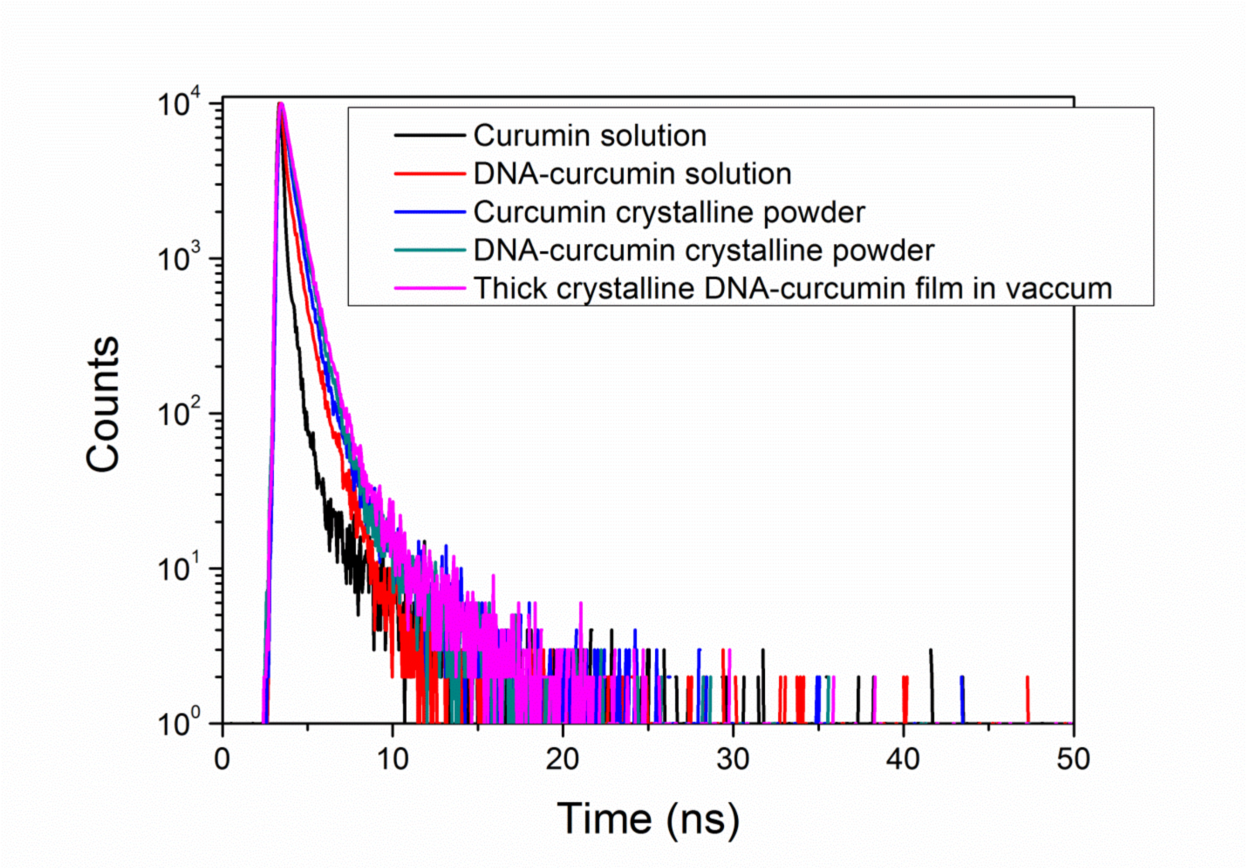

Supplementary Figure S4. Decay profiles of curcumin DNA-curcumin in solution, curcumin, DNA-curcumin in crystalline form, and thick crystalline DNA-curcumin film in vacuum conditions.

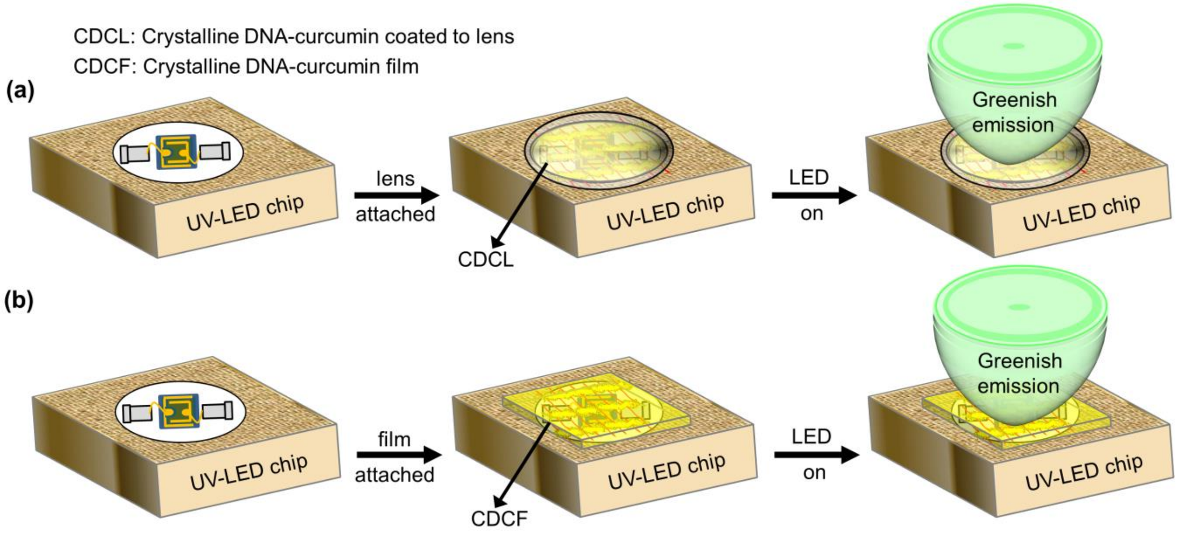

Supplementary Figure S5. Schematic graphical illustration of HyBi-LED process: crystalline DNA-curcumin. (**a**) Coated on lens. (**b**) Thick film–attaching techniques.

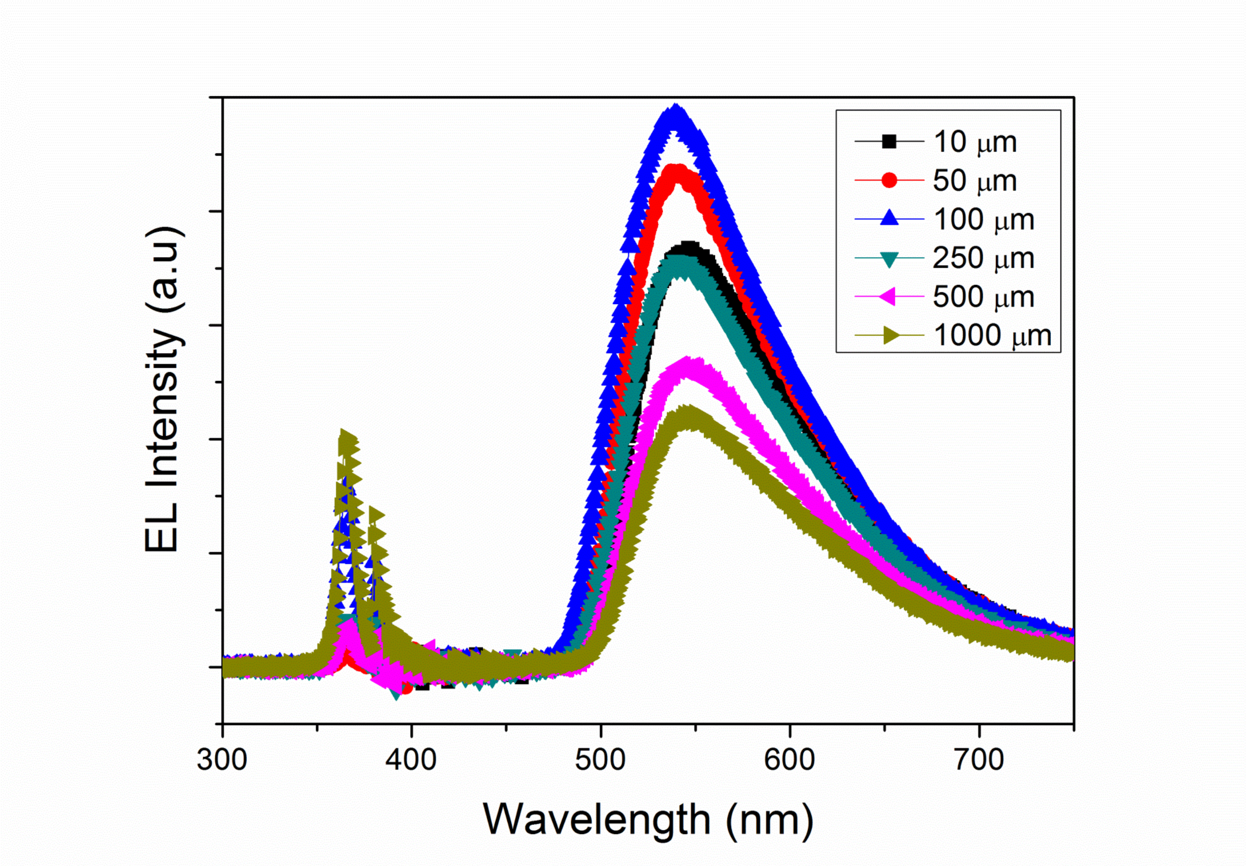

Supplementary Figure S6. Changes in the EL spectra of Bio-HLED (at injection current of 10 mA) with a different thickness of thick crystalline DNA-curcumin film.

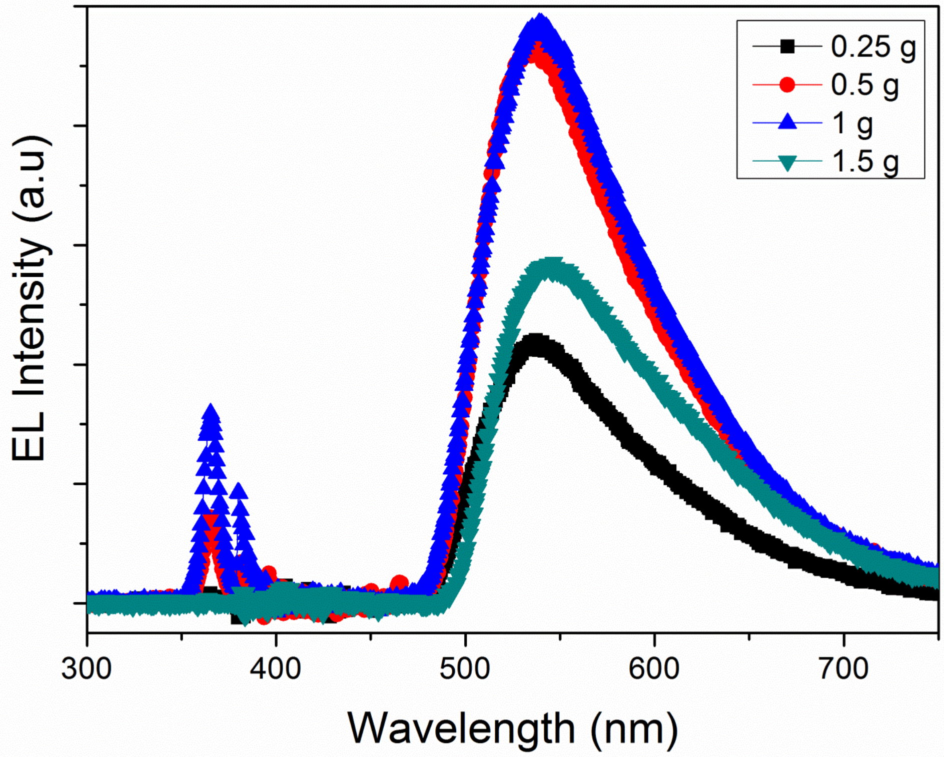

Supplementary Figure S7. Changes in the EL spectra of Bio-HLED with a different amount of curcumin to DNA at constant thickness of 100 μm thick crystalline DNA-curcumin film.

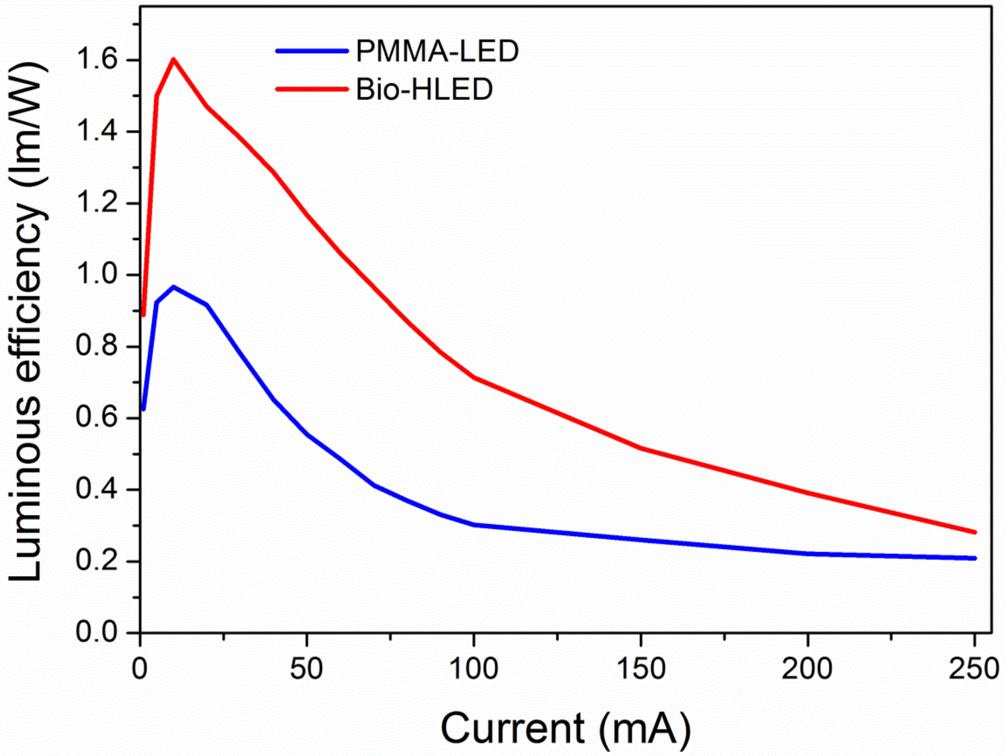

Supplementary Figure S8. Luminous efficiency including PMMA-LED comparison, which revealed that Bio-HLED showed bright luminescence from 1 to 250 mA compared to PMMA-LED.

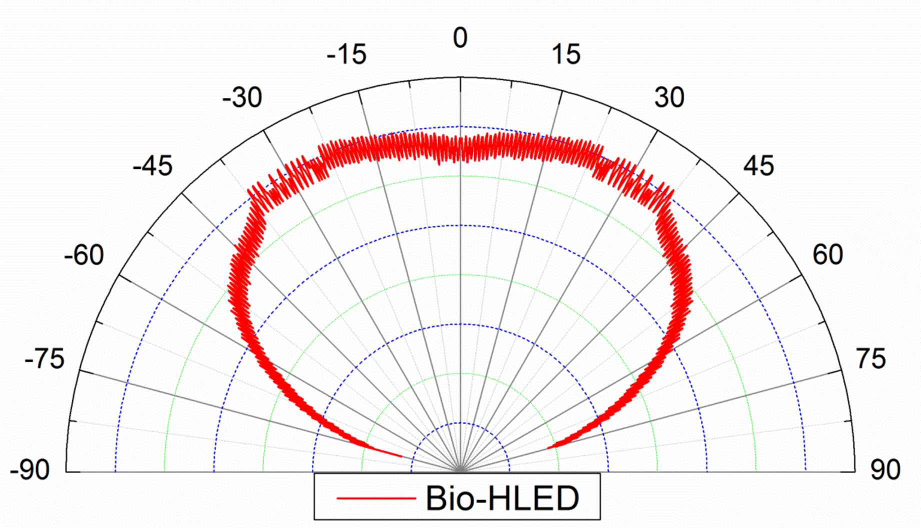

Supplementary Figure S9. The angular spatial photon distribution characteristics of the Bio-HLED.

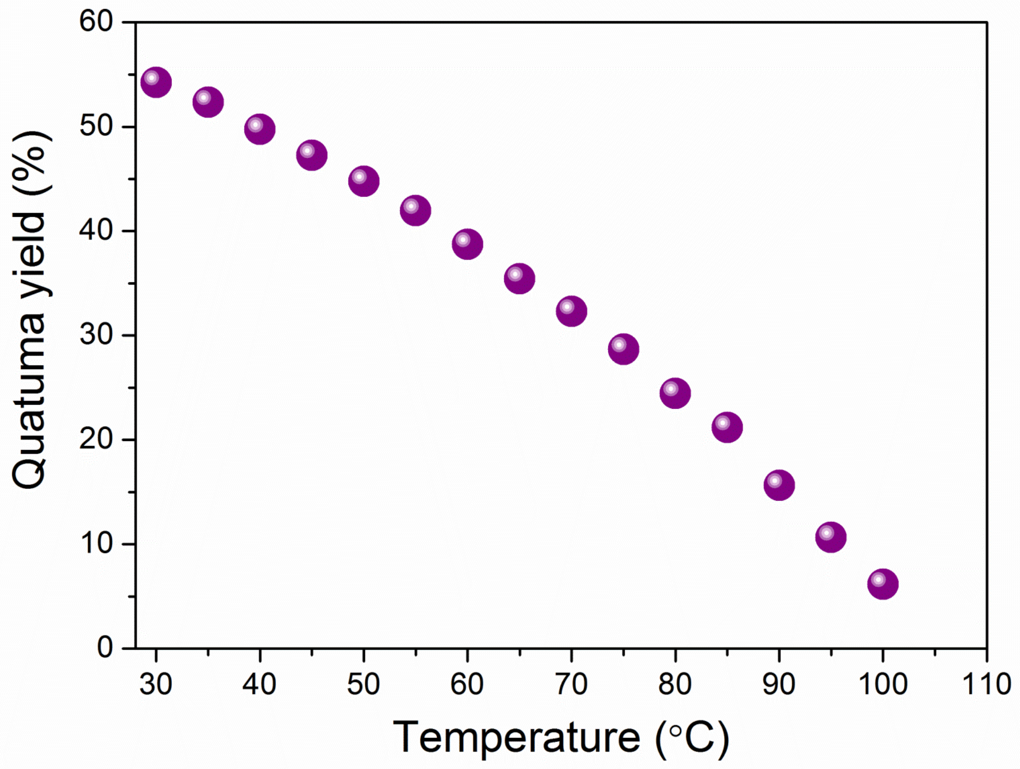

Supplementary Figure S10. The quantum yields of Bio-HLED as a function of temperature from 27 to 100 °C.

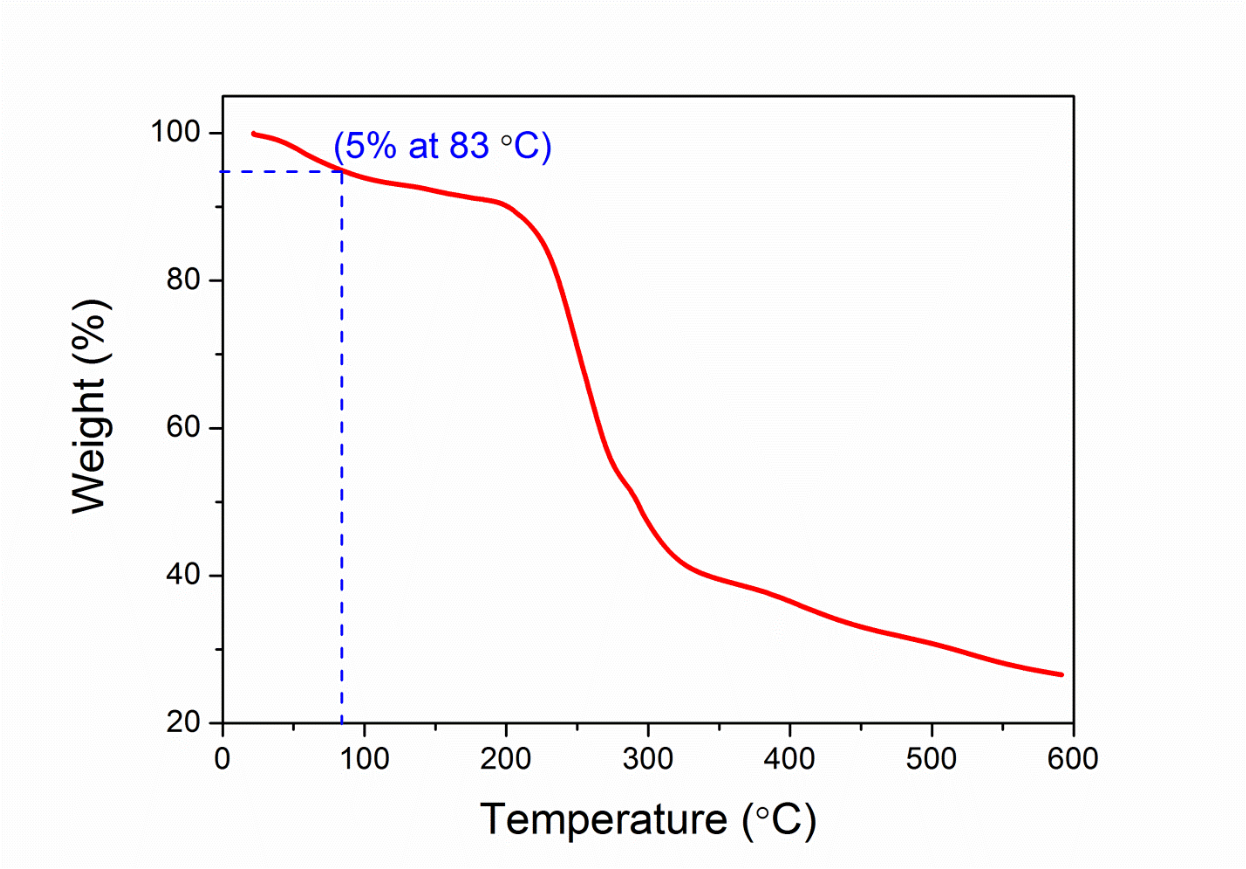

Supplementary Figure S11. TGA spectra of crystalline DNA-curcumin.

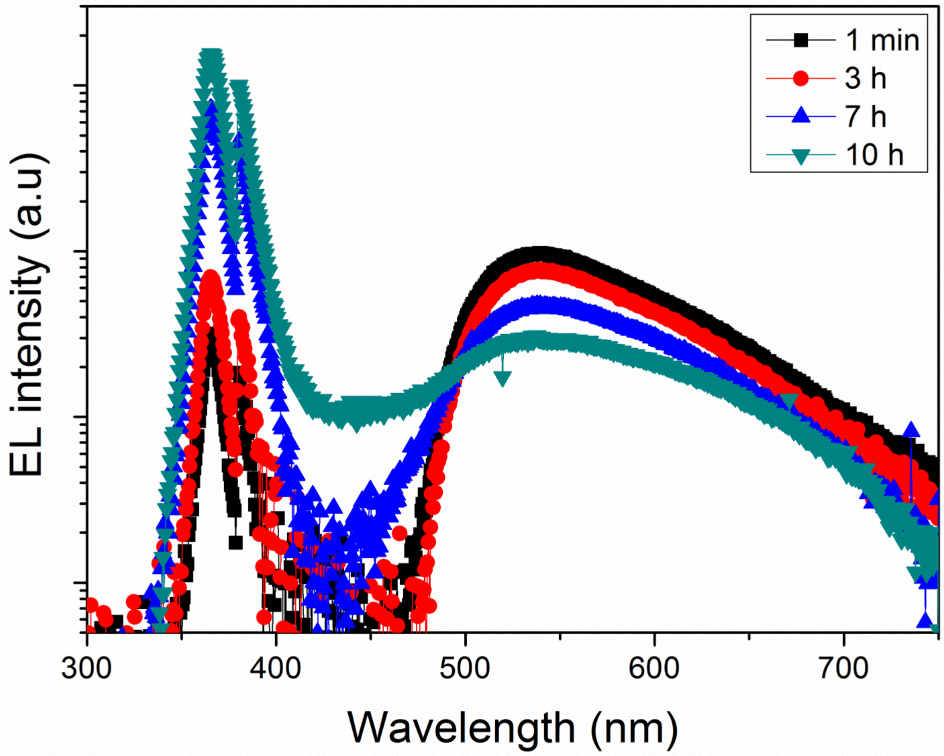

Supplementary Figure S12. Changes in the EL spectra of Bio-HLED (at injection current of 10 mA) over time.

Supplementary Table 1. Luminous efficiency and CIE colour co-ordinates of Bio-HLED based on different thickness of thick crystalline DNA-curcumin film measured at 10 mA.

**Thickness (μm)**	**Luminous efficiency (lm/W)**	**CIE colour co-ordinates (x,y)**
10	1.254	(0.40, 0.57)
50	1.508	(0.40, 0.56)
100	1.602	(0.39, 0.56)
250	1.426	(0.41, 0.56)
500	1.348	(0.42, 0.55)
1000	1.166	(0.43, 0.55)

Supplementary Table 2. Luminous efficiency and CIE colour co-ordinates of Bio-HLED based on different amount of curcumin to DNA at constant thickness of 100 μm thick crystalline DNA-curcumin film.

**Weight amount (g)**	**Luminous efficiency (lm/W)**	**CIE colour co-ordinates (x,y)**
0.25	1.288	(0.36, 0.56)
0.5	1.498	(0.38, 0.56)
1	1.602	(0.39, 0.56)
1.5	0.914	(0.43, 0.54)
